# Inhibition of Ca^2+^ entry by capsazepine analog CIDD-99 prevents oral squamous carcinoma cell proliferation

**DOI:** 10.3389/fphys.2022.969000

**Published:** 2022-09-15

**Authors:** Yuyang Sun, Emily K. Zboril, Jorge J. De La Chapa, Xiufang Chai, Viviane Nascimento Da Conceicao, Matthew C. Valdez, Stanton F. McHardy, Cara B. Gonzales, Brij B. Singh

**Affiliations:** ^1^ Department of Periodontics, School of Dentistry, University of Texas Health San Antonio, San Antonio, TX, United States; ^2^ Department of Comprehensive Dentistry, School of Dentistry, University of Texas Health San Antonio, San Antonio, TX, United States; ^3^ Department of Chemistry and the Center for Innovative Drug Discovery, University of Texas at San Antonio, San Antonio, TX, United States

**Keywords:** SOCE channels, Ca^2+^ homeostasis, TRPC1/STIM1, ER stress, mitochondrial dysfunction, and oral cancer

## Abstract

Oral cancer patients have a poor prognosis, with approximately 66% of patients surviving 5-years after diagnosis. Treatments for oral cancer are limited and have many adverse side effects; thus, further studies are needed to develop drugs that are more efficacious. To achieve this objective, we developed CIDD-99, which produces cytotoxic effects in multiple oral squamous cell carcinoma (OSCC) cell lines. While we demonstrated that CIDD-99 induces ER stress and apoptosis in OSCC, the mechanism was unclear. Investigation of the Bcl-family of proteins showed that OSCC cells treated with CIDD-99 undergo downregulation of Bcl-XL and Bcl-2 anti-apoptotic proteins and upregulation of Bax (pro-apoptotic). Importantly, OSCC cells treated with CIDD-99 displayed decreased calcium signaling in a dose and time-dependent manner, suggesting that blockage of calcium signaling is the key mechanism that induces cell death in OSCC. Indeed, CIDD-99 anti-proliferative effects were reversed by the addition of exogenous calcium. Moreover, electrophysiological properties further established that calcium entry was via the non-selective TRPC1 channel and prolonged CIDD-99 incubation inhibited STIM1 expression. CIDD-99 inhibition of calcium signaling also led to ER stress and inhibited mitochondrial complexes II and V *in vitro*. Taken together, these findings suggest that inhibition of TRPC mediates induction of ER stress and mitochondrial dysfunction as a part of the cellular response to CIDD-99 in OSCC.

## Introduction

Cancer is the second leading cause of death in the United States. Carcinomas are epithelial cell malignancies, and account for roughly 90% of reported cancers worldwide (Siegel et al., 2019). Squamous cell carcinoma (SCC) accounts for nearly 90% of all diagnosed head and neck cancers, with the majority of these tumors being found in the oral cavity, termed oral squamous cell carcinoma (OSCC) (Rivera, 2015a). More than 54,000 Americans are diagnosed with oral cavity or oropharyngeal cancer each year resulting in over 11,000 deaths, which equates to more than one death per hour, every hour (Siegel et al., 2021). Roughly 70% of patients diagnosed with OSCC have advanced disease at the time of diagnosis resulting in a 5-year survival rate as low as 38%, a number that has not changed in decades (Tsai et al., 2021). Current treatment approaches to OSCC include a combination of surgery, radiation, and coadjutant therapies (with paclitaxel and platinum-based drugs such as cisplatin) (Rivera, 2015b). Side effects of these treatments can be dramatic and include nerve and liver toxicity, gastrointestinal distress, and hemolytic anemia (Astolfi et al., 2013). Therefore, further studies and exploration of more efficacious treatment methods, which could result in fewer side effects, are imperative.

The transient receptor potential (TRP) superfamily is comprised of membrane bound cation channels that are responsible for a wide range of cellular functions. They are usually gated and transduce signals from outside the cell and induce a response via cation signaling. Many TRP channels play a role in cancer proliferation and metastasis (Adams et al., 2013; Samanta et al., 2018). Capsaicin (CAP) is known to activate the transient receptor potential cation channel subfamily V member 1 (TRPV1) channels and inhibit cancer growth (Clark and Lee, 2016). Additionally, capsazepine (CPZ), is also capable of sensitizing OSCC cells and other cancer types to apoptosis (Gonzales et al., 2014; Yang et al., 2019), making it an ideal candidate for the inhibition of cancer cell growth. Our group previously determined that CAP and CPZ have anticancer activity via a different mechanism that does not involve TRPV1 (Gonzales et al., 2014; De La Chapa et al., 2019a). Indeed, both CAP and CPZ induce high levels of reactive oxygen species (ROS), disruption of the mitochondrial membrane potential, and apoptosis selectively in cancer cells (Ziglioli et al., 2009). This key discovery led us to develop a safer and more potent new class of anticancer drugs based on the CPZ pharmacophore (now entitled 1,3-thioureas). This new class of drugs acts independently of TRPV1 thereby eliminating TRPV1-related adverse effects (e.g., thermal dysregulation). To accomplish this, we generated more than 60 novel CPZ analogs, in order to eliminate TRPV1 activity and improve anti-proliferative effects, with CIDD-99 being most effective (De La Chapa et al., 2019b). Although it is clear that CIDD-99 preferentially kills OSCC over normal oral keratinocytes, its mechanism is not yet identified. Previous reports have demonstrated that CAP treatment inhibited electron transport resulting in ROS production, disruption the mitochondrial membrane potential, and ultimately resulting in apoptosis in pancreatic cancer cells (Pramanik et al., 2011). Importantly, vanilloids, like CAP, are capable of blocking the transfer of electrons from complex I of the electron transport chain to Coenzyme Q (CoQ), which leads to the production of ROS and ultimately to apoptosis (Ziglioli et al., 2009). Moreover, addition of antioxidant rescues the effects of CIDD-99 (De La Chapa et al., 2019a). Similarly, polygodial, another TRPV1 agonist, has been shown to block mitochondrial ATP synthase in yeast (Lunde and Kubo, 2000; Castelli et al., 2005; De La Chapa et al., 2018).

The other possibility could be that CIDD-99 induces ER stress in OSCC cells leading to apoptosis. Intracellular calcium is implicated as a key player in CAP-induced cell death, where, CAP induces upregulation of ER stress markers GRP78 (BIP), CHOP, and IRE1 (Huang et al., 2009; Lee et al., 2009). Likewise, we demonstrated that CIDD-99 induces ER stress, but the mechanism is not clear. Our previous studies showed that transient receptor potential canonical 1 (TRPC1) plays a key role in ER stress (Da Conceicao et al., 2019; Sukumaran et al., 2019). TRPC1 is stimulated by both calcium store-depletion as well as by the activation of G-protein coupled receptors and increases calcium entry in both excitable and non-excitable cells (Clapham et al., 2005). The endogenous Ca^2+^ entry channel is dependent on the Orai1–TRPC1–STIM1 complex, and the presence of ER stressors decreased expression of TRPC1, Orai1 and STIM1 (Da Conceicao et al., 2019). Recent studies have also suggested a functional role of TRPC1 in various cancers (Elzamzamy et al., 2020; Zhang et al., 2020; Zeng et al., 2021). Thus, in this study we elucidated the role of CIDD-99 in ER stress and apoptosis of OSCC cells. Our data shows that ER stress and calcium homeostasis disruption is caused by CIDD-99. Importantly, CIDD-99 inhibited TRPC1 function resulting in decreased levels of ER calcium and in the induction of ER stress. Moreover, STIM1 expression was downregulated in the presence of CIDD-99, in a concentration dependent manner. Overall, our data suggest that CIDD-99 triggers apoptosis via an ER stress-mediated cell death pathway triggered by the inhibition of TRPC1 channels.

## Materials and methods

### Human OSCC cell lines

Cal-27 cells were obtained from ATCC (Manassas, VA, United States). HSC-3 cells were provided by Dr. Brian Schmidt at New York University College of Dentistry. Cells were maintained in DMEM (Gibco, Carlsbad, CA) containing 10% FBS at 37 °C in 5% CO_2_. All experiments were performed within 5–10 passages of both Ca-27 and HSC-3 as described in (De La Chapa et al., 2018; De La Chapa et al., 2019b).

### Cell viability assay

Cell viability was measured by 3-[4,5-dimethylthiazol-2-yl]-2,5-diphenyltetrasolium bromide (MTT) (Sigma-Aldrich, St Louis, MO, United States) according to the manufacturer’s protocol. Cells were seeded in 96‐well plates at a density of 1 × 10^4^ cells/well. The cultures were grown for 24 h followed by the addition of fresh serum free medium 24 h prior to the experiment. Cells were treated with varying concentrations of CIDD-99, and 10 μL of MTT reagent (5 mg/ml MTT in PBS) was added to each well and incubated in a CO_2_ incubator for 4 h. The resulting formazan dye was solubilized in 50 μL of DMSO. Absorbance was measured at a test wavelength of 570. Cell viability was expressed as a percentage of the control culture.

### Western blotting analysis

Cal-27 cells were treated for 24 h with vehicle control and CIDD-99 at the indicated concentrations and then harvested and lysed in Laemmli Lysis Buffer. Cell lysate concentration was determined by A570 reading using Pierce BCA protein assay (Thermo Fisher Scientific). Cell lysates containing equal concentration of protein (35 μg) were separated using electrophoresis in NuPAGE Novex 4–12% BisTris gels. Separated proteins were transferred to PVDF membrane and membrane was blocked in 5% milk solution. Appropriate primary antibodies (Cell Signaling, MA, United States) were incubated overnight. After washing with PBST, secondary antibodies (Cell Signaling, MA, United States) were applied and detected by the Clarity Western ECL Substrate and Clarity Max Western ECL Substrate (Bio-Rad Laboratories, Hercules, CA, United States). Analysis and results were corrected for protein loading by normalization for β-actin (Cell Signaling, 4970S) expression. Densitometric analysis was performed using Fiji analysis.

### Mitro-tracker red staining

Cells were seeded in 35 mm glass bottom dishes and allowed to grow overnight in phenol red free DMEM with 10% FBS. The following day, cells were treated with 10 μM CIDD-99 and a vehicle control and incubated for 2 h. Cells were then exposed to Mito Tracker Red dye (ThermoFisher) at a final concentration of 125 nM and Hoechst 33342 (1 μg/ml) and incubated at 37°C for 30 min. Cells were then washed and imaged using a confocal microscope.

### MitoCheck® complex II and V assay

Mitocheck® Assays (Caymen Chemicals, Ann Arbor, MI) were used to assess the effect of CIDD-99 on mitochondrial complexes. Bovine heart mitochondria are provided in this kit. Mitochondria were treated with CIDD-99 at 5 and 100 μM concentrations and rates of inhibition for complex II and V were assessed according to the manufacturer’s protocol.

### Calcium imaging

Cal-27 cells were incubated with 2 μM fura-2 (Molecular Probes) for 45 min and then washed three times with Ca^2+^ free SES buffer. Cells were monitored post-treatment with a CCD camera-based imaging system. The images of multiple cells collected at each excitation wavelength were processed using the C imaging, PCI software (Compix Inc., Cranberry, PA), to provide ratios of Fura-2 fluorescence from excitation at 340 nm to that from excitation at 380 nm (F340/F380) as described in (Da Conceicao et al., 2019; Sukumaran et al., 2019).

### Patch clamp Assay

Coverslips with Cal-27 cells previously treated with varying concentrations of CIDD-99, or vehicle control were transferred to the recording chamber and perfused with an external Ringer’s solution of the following composition (mM): NaCl, 145; KCl, 5; MgCl_2_, 1; CaCl_2_, 1; Hepes, 10; Glucose, 10; pH 7.4 (NaOH) as described in (Da Conceicao et al., 2019). Briefly the patch pipette had resistances between 3–5 mV after filling with the standard intracellular solution that contained the following (mM): cesium methane sulfonate, 145; NaCl, 8; MgCl_2_, 10; Hepes, 10; EGTA, 10; pH 7.2 (CsOH). The currents were initiated by the addition of thapsigargin, and maximum peak currents were calculated at a holding potential of -80 mV. The I/V curves were made using a ramp protocol where current density was evaluated at various membrane potentials and plotted.

### Statistical analysis

Statistical analysis was performed using GraphPad Prism 8.3.0 (San Diego, CA, United States). Cell viability, western blot data and calcium imaging data were compared using an unpaired t-test and one-way ANOVA, with *p* < 0.05 showing significance. Data is presented in graphs representing mean ± SD.

## Results

### CIDD-99 shows antiproliferative effects in OSCC cell lines in a dose-dependent manner

**FIGURE 1:**
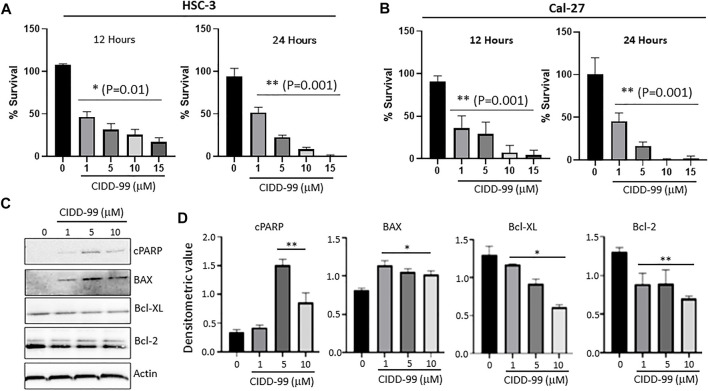
Cal-27 and HSC-3 cells show dose-dependent reduced proliferation when treated with CIDD-99. Cal-27 and HSC-3 OSCC cell lines were treated with various concentrations of CIDD-99 over varying time courses and percent survival was measured by MTT cell viability assay. **(A)** HSC-3 cells treated for 12 and 24 h. **(B)** Cal-27 cells treated for 12 and 24 h respectively. *n* = 3, with 3 independent replicates. Significance was determined using one-way ANOVA and * indicates *p* = 0.01, whereas ** indicates *p* = 0.001 respectively. **(C)** Cal-27 cells were treated with CIDD-99 at concentrations of 0, 1, 5 and 10 µM for a period of 24 h. After 24 h, cells were lysed and analyzed for apoptotic markers by western blot. Western blots were quantified using ImageJ. **(D)** Western blot antibody ratio for cPARP, BAX, Bcl-XL, and Bcl-2 compared to actin loading control. *n* = 3 with 2 independent replicates and significance was determined using T-test and * indicates *p* = 0.01, whereas ** indicates *p* = 0.001 respectively.

Our previous studies showed that the novel CPZ analog, CIDD-99, is a potent inducer of cytotoxic effects in multiple oral cancer cell lines. Additionally, CIDD-99 was effective at reducing tumor volumes in Cal-27-derrived xenografts (De La Chapa et al., 2019a). To determine if the cytotoxic effects of this drug were dose-dependent OSCC cells lines were treated with varying concentration of CIDD-99 and cells were analyzed for cell viability via MTT assays. In addition, we used two different cell lines; Cal-27 which represent capsular oral tumors, whereas HSC-3 represent the non-capsular tumors to see if CIDD-99 is affective in both types of oral cancers. CIDD-99 was able to induce cell death in a dose-dependent manner in both HSC-3 and Cal-27 oral cancer cell lines. Importantly, CIDD-99 was effective in both oral cancer cell lines in as little as 12 h (Figures 1A,B), and continues to decrease cell viability at 24 h, (Figures 1A,B). At 48 h, concentrations as low as 10 µM of CIDD-99 can reduce the culture to almost 0% viability when compared to the vehicle treated control cells, suggesting that CIDD-99 is significantly able to modulate cancer cell viability.

To determine the nature of the reduction in cell viability and to determine if cells were undergoing apoptosis, Bcl-family proteins and PARP cleavage were examined. Cal-27 cells that were treated with 0, 1, 5 and 10 µM of CIDD-99 (24 h) were used and whole protein lysates were analyzed via western blotting (Figures 1C,D). The results show that Bcl-XL, an antiapoptotic protein, was downregulated, and an inverse relationship was observed with increasing doses of CIDD-99 (Figures 1C,D). The same was true for Bcl-2, where expression of Bcl-2 was also significantly decreased in the presence of higher doses of CIDD-99 (Figures 1C,D). In contrast, the expression of proapoptotic protein BAX was upregulated in treatment groups compared to the vehicle control (Figures 1C,D), and that PARP was cleaved at significant levels at doses of 5 and 10 µM of CIDD-99 (Figures 1C,D). In contrast no change in actin levels were observed (Figures 1C,D), suggesting that the changes in protein expression responsible for the induction of apoptosis by CIDD-99 were specific. These expression patterns are consistent with apoptotic events observed above that further establishes that CIDD-99 induces apoptosis in oral cancer cells.

### CIDD-99 treatment results in the dysregulation of calcium homeostasis as well as inability to activate TRPC1

**FIGURE 2:**
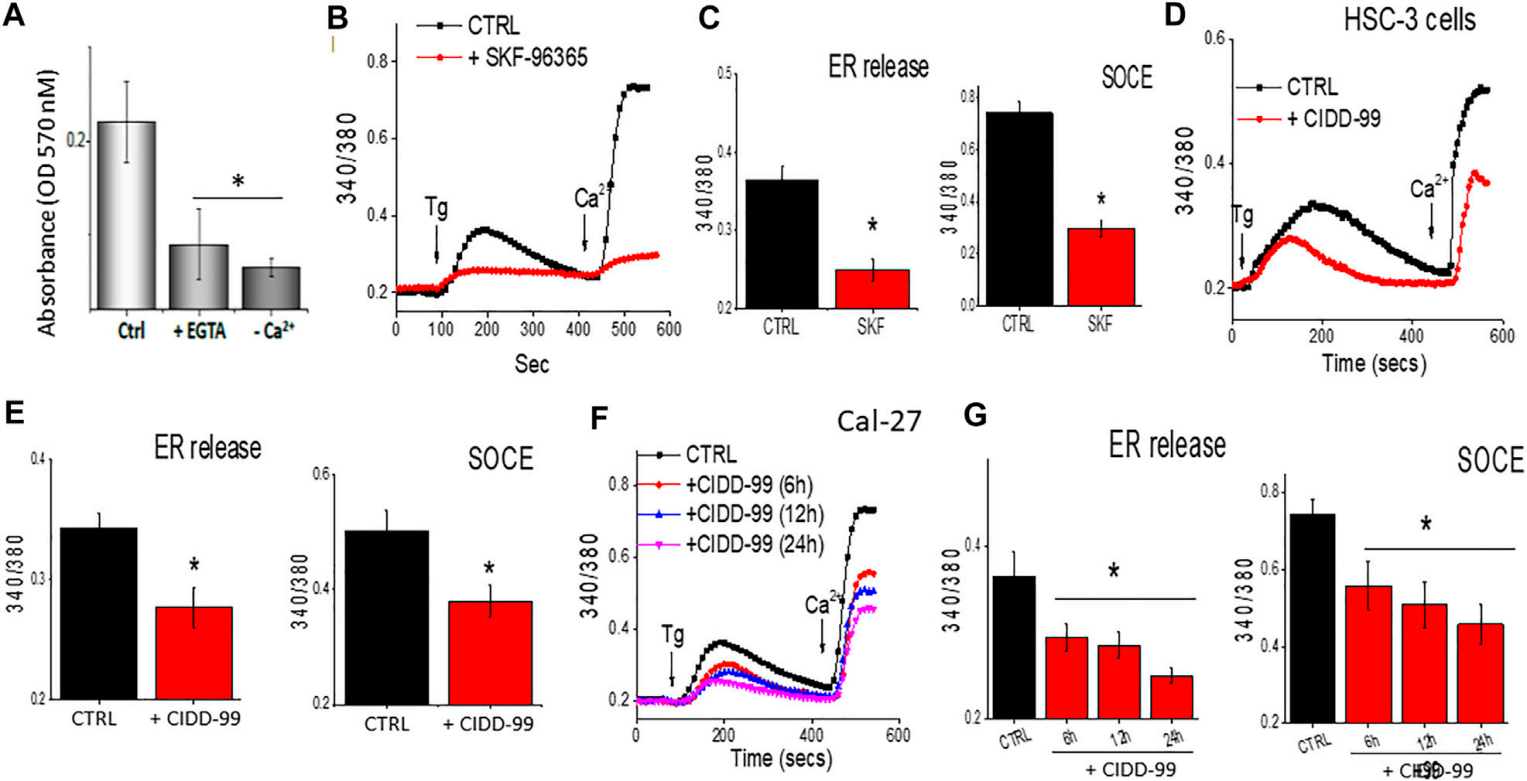
CIDD-99 disrupts calcium homeostasis in OSCC cell lines and reduces ability to activate TRPC1. **(A)** Cal-27 cells were treated with EGTA (5 mM) or medium devoid of external calcium for a period of 24 h and percent survival was measured by MTT assay. Significance was determined using one-way ANOVA and * indicates *p* = 0.05, n = 3 with 4 independent replicates. **(B)** Cal-27 cells were treated with SKF-96365 (10 µM) or control untreated cells for 24 h, and fura 2 analog plots (340/380 ratio) were measured in the presence of 2 μM Thapsigargin. **(C)** Quantification of fluorescence ratio (peak value) from 60–80 individual cells is shown as bar graph. * Indicates data that is significantly different from control vehicle treated cells (*p* < 0.05 using one-way ANOVA). **(D)** HSC-3 cells were treated with CIDD-99 (10 μM) with different hours and fura 2 analog plots (340/380 ratio) were measured. **(E)** Quantification of fluorescence ratio (peak value) from 70–90 individual cells is shown as bar graph. * Indicates significantly different from control *p* < 0.05 (using one-way ANOVA). **(F)** Cal-27 cells were treated with CIDD-99 (10 μM) for different time-points and Fura 2 analog plots (340/380 ratio) were measured. **(G)** Quantification of fluorescence ratio (peak value) from ∼100 cells are shown as bar graph and significance (*p < 0.05) was estimated using one-way ANOVA.

One of the main factors that modulates cell proliferation in oral cancer cells is cytosolic calcium levels (Cui et al., 2017). Decreasing external calcium levels, had a negative effect on cell survival and both addition of 5 mM EGTA, that chelates calcium, or cells that were devoid of external calcium, showed an increase in cell death in OSCC cells (Figure 2A). In addition, calcium imaging was evaluated, which was stimulated by the administration of Thapsigargin (Tg), a SERCA pump inhibiter that causes depletion of ER calcium stores, essential for the activation of calcium entry (Jaskulska et al., 2020), to determine if loss of calcium entry was observed upon the addition of CIDD-99. Addition of SKF-96365, which is a non-specific Ca^2+^ channel blocker (Selvaraj et al., 2012), also decreased Ca^2+^ entry in OSCC cells (Figures 2B,C). In addition, treatment using a selective Orai1 inhibitor Synta-66 also decreased calcium entry in these cells (data not shown). Similarly, our results also showed that addition of CIDD-99 in HSC-3 cells for 12 h had a significant decrease in calcium entry, along with a decrease in ER calcium levels (Figures 2D,E). Importantly, Cal-27 cells pre-treated with CIDD-99 showed a time dependent decrease in calcium entry. Also observed was a higher loss of both ER calcium as well as calcium entry in cells that were treated with 5 µM of CIDD-99 (Figures 2F,G). Together these results suggest that OSCC cells treated with CIDD-99 were unable to restore calcium homeostasis. Finally, both calcium entry as well as ER and intracellular calcium levels were decreased, which can result in cell death.

**FIGURE 3:**
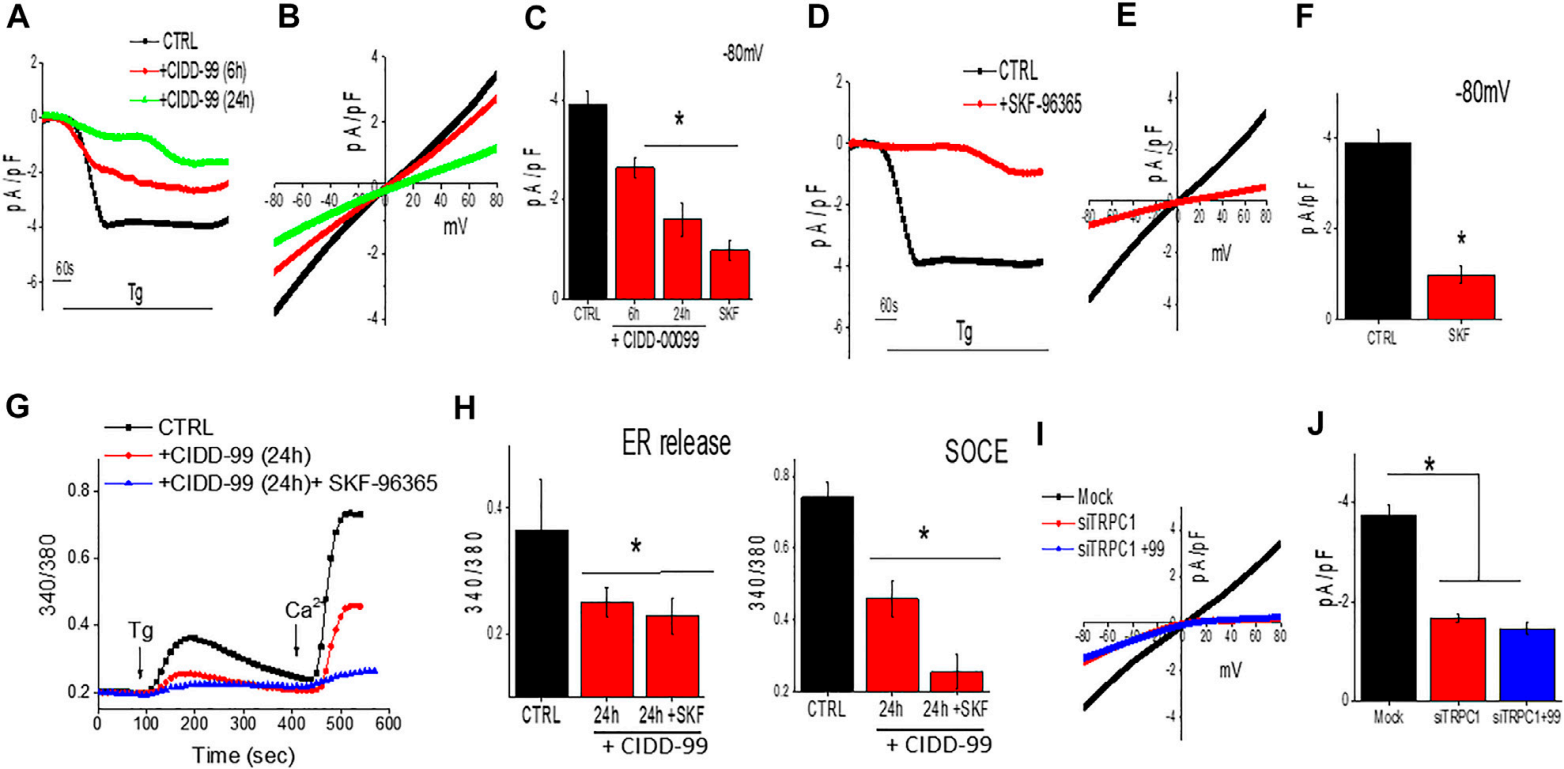
Cells treated with CIDD-99 inactivate TRCP1 channels. **(A)** Representative traces showing inward currents (at holding potential of -80 mV) upon application of Thapsigargin (2 µM) in the bath solution from Cal-27 cells and with CIDD-99 pretreated cells. **(B)** Relative IV curves (acquired when currents reach peak) are shown. **(C)** Quantification of current density under these conditions are shown from 7–9 cells for each condition. * Indicates significantly different (using one-way ANOVA) from control *p* < 0.05. **(D)** Representative traces showing application Thapsigargin (2 µM) in bath solution induced inward currents with holding potential of -80 mV from Cal-27 cells and SKF-96365 (10 µM) pretreated cells. **(E)** Relative IV curves (acquired when currents reach peak) are shown. Quantification of current intensity (using one-way ANOVA, n = 10–12 cells) under these conditions are shown as bar graph **(F)**. **(G)** Fura 2 analog plots (340/380 ratio) under various conditions as labeled were measured. Quantification of fluorescence ratio (peak value) from 50–80 cells is shown as bar graph **(H)** * Indicates significantly different (*p* < 0.05 using one-way ANOVA) from control untreated cells. Individual showing IV curves in controlsiRNA (Mock), TRPC1siRNA (siTRPC1) and TRPC1siRNA + CIDD-99 **(I)**. Quantification of current intensity (peak value) is shown as bar graph **(J)**. * Indicates significantly different (*p* < 0.05 using one-way ANOVA) from control Mock treated cells.

To further establish the molecular identity of the calcium entry channel, whole-cell current recordings were performed in oral cancer cells. Addition of Tg (store depletion) in Cal-27 cells induces an inward current which was non-selective in nature and reversed between 0 and −5 mV (Figures 3A–C). Importantly, the channel properties were similar as those previously observed with TRPC1 channels (Sukumaran et al., 2016). Furthermore, addition of CIDD-99, also decreased Ca^2+^ entry in Cal-27 cells in a time dependent manner, without altering the channel properties (had the same revere potential) (Figures 3A–C). The addition of SKF-96365, also decreased Ca^2+^ entry in Cal-27 cells (Figures 3D,E). Similar results were also observed with HSC-3 cells; a decrease in non-selective calcium currents was observed, which was again decreased by the addition of CIDD-99 (data not shown). Co-treatment with CIDD-99 and SKF-96365 also produced reduction in ability to restore ER calcium homeostasis and a further decrease in calcium entry (Figures 3F,G), suggesting that, similar to SKF-96365, CIDD-99 is also selective for inhibiting the calcium entry channels (Figures 3D–F). Interestingly, co-treatment with CIDD-99 and SKF-96365 was more potent than CIDD-99 alone. Furthermore, silencing of oral cancer cells with TRPC1siRNA also showed a decrease in the calcium currents, which was not further decreased by the addition of CIDD-99 (Figures 3I,J). Together these results suggest that TRPC1 could contribute to the endogenous Ca^2+^ entry channels in OSCC cells and calcium currents by TRPC1 channels are specifically inhibited by the addition of CIDD-99.

### CIDD-99 prevents cancer cell migration and prolonged treatment decreases STIM1 expression

**FIGURE 4:**
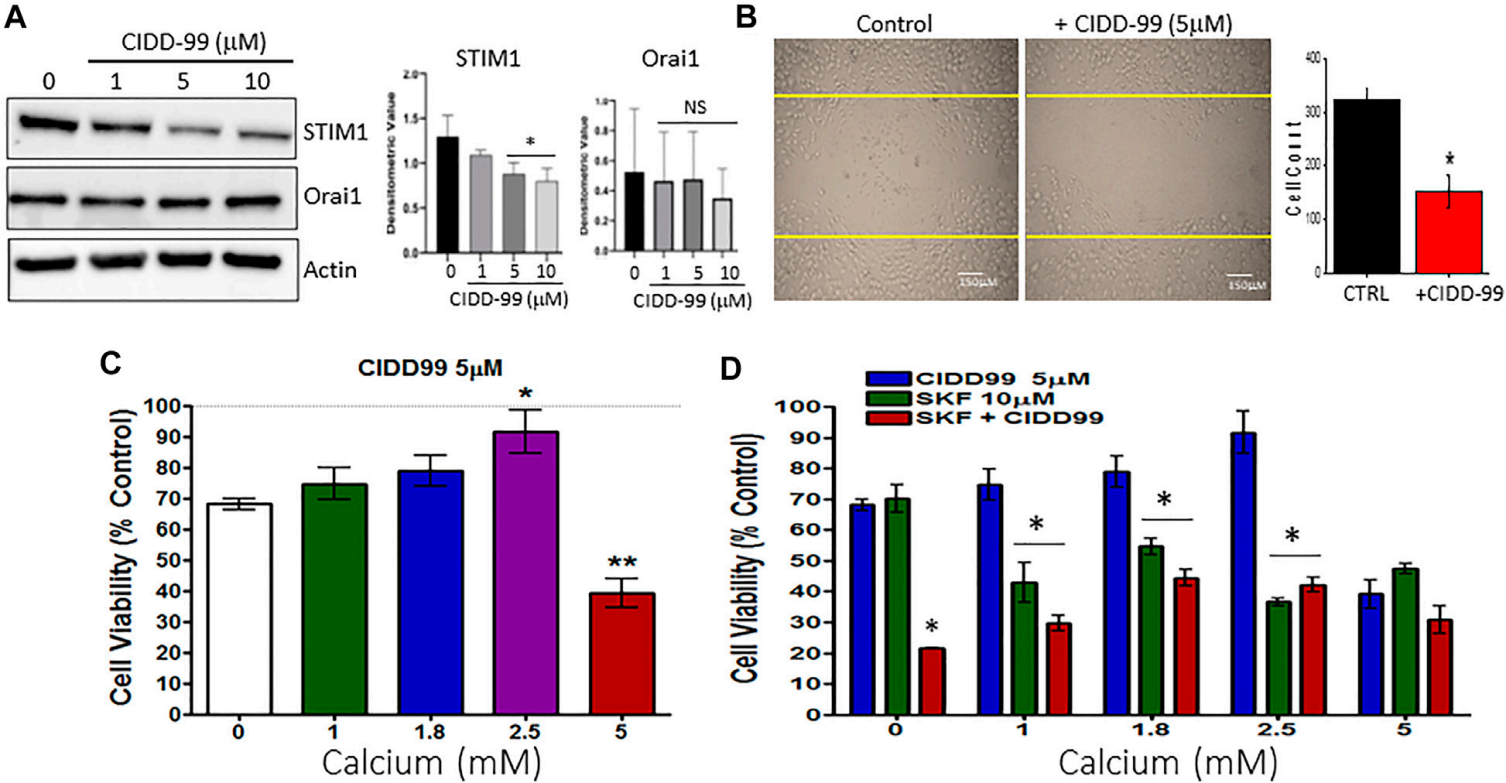
OSCC cells treated with CIDD-99 show downregulation of STIM1 and increase cell migration. **(A)** Cal-27 cells were treated with CIDD-99 at concentrations of 0, 1, 5 and 10 µM for a period of 24 h. After 24 h, cells were lysed and analyzed for calcium channel proteins, STIM1 and ORAI1. Quantification (*n* = 3) is shown as bar graph (mean ± SD) on the right. * Indicates significantly different (T-test) from control untreated *p* < 0.05, NS indicates no significant difference. **(B)** Cell migration assays were performed where cells were grown for 24 h scratched using a pipette with and without required CIDD-99 and images were taken after 24 h using a ×20 objective. Quantification is shown as bar graph, where * indicates significance (*p* < 0.05, using one-way ANOVA, *n* = 4) from un-treated control. **(C)** Cell viability assay of Cal-27 cells treated with 5 µM CIDD-99 and increasing calcium concentrations for 24h; *p < 0.05, and **p < 0.001 (using one-way ANOVA, *n* = 4 with 3 independent replicates). **(D)** Cell viability assay of Cal-27 cells treated with 5 µM CIDD-99 and in combination with 10 µM SKF and increasing calcium concentrations. Bar graph shows quantification (*n* = 4 with 3 independent replicates) and * indicates significance (*p* < 0.05 using one-way ANOVA) when compared with CIDD-99 alone.

The data presented thus far indicates that the inhibition of TRPC1 by CIDD-99 reduces the ability of OSCC cells to maintain calcium homeostasis. Thus, we next evaluated the expression of proteins that are important for TRPC1 regulation. Importantly, Cal-27 cells were treated with 0, 1, 5 and 10 µM of CIDD-99 for 24 h and whole protein lysates were analyzed via western blot. Groups treated with 5–10 µM of CIDD-99, showed that CIDD-99 downregulation of STIM1, while ORAI1 expression was unchanged (Figure 4A). Similarly, no change in control actin levels were observed, indicating that loss of STIM1 expression may be the reason for the decrease in calcium entry in CIDD-99 treated cells. Oral cancer cells are also highly metastatic, thus, we next evaluated if CIDD-99, prevents cancer cell migration using the wound-healing assay. As shown in Figure 4B, control untreated cells showed a modest increase in cell migration within 24 h. Importantly, oral cancer cells that were treated with CIDD-99 (5 μM) showed a significant decrease in cell migration (Figure 4B). To determine if the effects of CIDD99 are reversible, we increased external calcium levels. Supplementation with 2.5 mM calcium resulted in a complete restoration of cell viability (Figure 4C). However, further increase (5 mM external calcium) significantly inhibited cell survival. Importantly, co-treatment with SKF-96365 and CIDD-99 also had a synergistic effect, in inhibiting cancer cell proliferation that was not reversed with the addition of exogenous calcium (Figure 4D). Together these results show that loss of calcium homeostasis is the critical factor that is important for oral cancer cell survival and its migration.

### CIDD-99 treatment induces upregulation of ER stress markers and mitochondrial dysfunction

**FIGURE 5:**
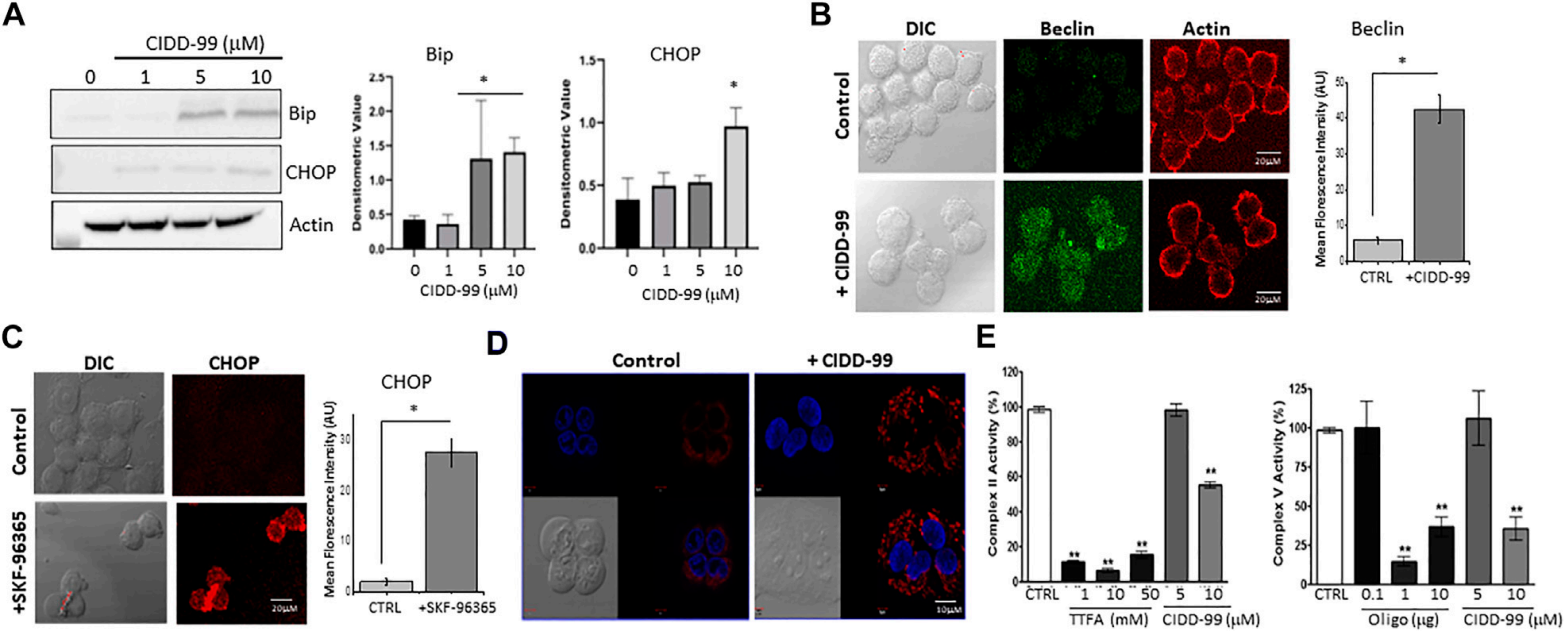
OSCC cells treated with CIDD-99 suffer ER stress and mitochondrial disfunction. **(A)** Cal-27 cells were treated with CIDD-99 at various concentrations as labeled for 24 h, lysed, and analyzed for ER stress markers. Quantification (*n* = 3) of Bip is shown as bar graph and * indicates significantly different (using one-way ANOVA) from control untreated cells *p* < 0.05. **(B)** Representative confocal images of Cal-27 cells showing expression of Beclin-1 in control and CIDD-99 treated cells, actin was used as control. Bar graph shows quantification and * indicates significance (*p* < 0.01 using one-way ANOVA) when compared with control. **(C)** Confocal images showing CHOP expression in control and SKF-96365 treated Cal-27 cells, bar graph represents quantification, * indicates significance (*p* < 0.01 using one-way ANOVA) when compared with control untreated. **(D)** Mito-tracker red staining shows mitochondrial morphological changes when treated with CIDD-99 for 2 h compared to the vehicle control. Mitochondrial activity was analyzed using Mitocheck assay showing the effect of CIDD-99 on complex II and complex V **(E)** in bovine cardiac mitochondria. Bar graph shows quantification and ** indicates significance (*p* < 0.01 using one-way ANOVA) when compared with control.

Addition of CIDD-99 leads to a decrease in calcium homeostasis, which decreases OSCC cell viability and migration; however, the mechanism is not clear. It has been established that CPZ analogues upregulates ER stress markers (Oh and Lim, 2009), and as ER calcium levels were decreased, it is possible that CIDD-99 induces an ER stress-driven apoptotic pathway. Importantly, addition of CIDD-99 leads to an increase in ER stress proteins. As expected, a gradual increase in BIP protein was observed with higher doses of CIDD-99 (Figure 5A). Similarly, an increase in CHOP protein was also observed, whereas no change in actin levels was observed (Figure 5A). Consistent with these results an increase in Beclin-1 expression was observed in cells that were treated with CIDD-99 and a diffused beclin-1 staining was observed in cells that were treated with CIDD-99 (Figure 5B). Similarly, treatment with SKF-96365 also showed an increase in CHOP levels (Figure 5C), suggesting that loss of calcium homeostasis leads to ER stress, which could further prevent cell survival of oral cancer cells.

Calcium entry has also been shown to be important for mitochondrial function. Importantly Cal-27 cells showed normal mitochondrial morphology in untreated conditions (Figure 5D). However, when treated with CIDD-99 for 2 h the microtubule network begins to deteriorate resulting in punctiform morphology, which is a hallmark of early apoptosis (Karbowski and Youle, 2003). Oligomycin (Oligo) and Thenoyltrifluoroacetone (TTFA), which are inhibitors for complex V and complex II were used as positive controls that showed inhibition of mitochondrial activity. Additionally, both complex II activity of the electron transport chain, as well as ATP synthase (complex V) activity was decreased when treated with CIDD-99 (Figure 5E). Conjointly, these results suggest that CIDD-99 induced loss of calcium homeostasis is key in modulating ER stress and mitochondrial dysfunction, which leads to the induction of apoptosis required for the demise of oral cancer cells upon treating with CIDD-99.

## Discussion

Oral cancer patients generally have a poor prognosis, with only about 66% of patients surviving 5-years after diagnosis (Tsai et al., 2021). Treatments for oral cancer also have many adverse side effects thereby limiting its use (Astolfi et al., 2013; Gonzales et al., 2014). We developed CIDD-99 based on the CPZ pharmacophore, which showed that CIDD-99 produced cytotoxic effects in cancer cell lines and had an anti-tumor effect in Cal-27-derived xenografts (De La Chapa et al., 2019a; De La Chapa et al., 2019b). CIDD-99 also showed no adverse effects on healthy control cells at doses used in treatment and produced no adverse effects in rat models of orofacial pain (De La Chapa et al., 2019a). We also demonstrated that this molecule acts independently of TRPV1, and produced ROS, possibly leading to apoptosis (De La Chapa et al., 2019b), but the mechanism was not clear. Accumulating evidence have demonstrated that intracellular calcium homeostasis, especially alteration in calcium signaling is involved in tumor initiation, progression, and metastasis (Kania et al., 2015; Patergnani et al., 2020); thus, targeting calcium signaling could be critical for cancer therapy. Our results show that calcium homeostasis was important for the survival of oral cancer cells and addition of CIDD-99 was able to prevent calcium entry, thereby inducing cell death in oral cancer cells. We further explored the manner by which, CIDD-99 produces cytotoxic effects in oral cancer cells. We first determined that CIDD-99 reduces cell viability in a dose- and time-dependent manner. Importantly, Bcl-family proteins Bcl-2 and Bcl-XL that are anti-apoptotic proteins (Sukumaran et al., 2016) were downregulated, whereas pro-apoptotic protein Bax and cleaved PARP were upregulated upon the addition of CIDD-99. ER Ca^2+^ release has been shown to be critical for Ca^2+^ signaling network and regulates fundamental cellular functions, such as cell proliferation (Kania et al., 2015). Additionally, we established that CIDD-99 not only decreased, calcium entry, but also decreased ER calcium levels, which induces ER stress that can modulate apoptosis in oral cancer cells. Interestingly, mitochondrial function was also inhibited by the addition of CIDD-99, which can again be due to the loss of calcium homeostasis, as recent studies have shown that mitochondria and ER crosstalk determine the cell fate by modulating mitochondrial Ca^2+^ homeostasis and metabolism (Missiroli et al., 2017; Bustos et al., 2021).

To further explore the molecular identity of the calcium entry channel that is modulated by CIDD-99, whole cell calcium currents were evaluated on cells treated with CIDD-99. Our data suggest that inhibition of TRPC1 as a possible explanation for this effect, as TRPC1 activity is diminished following treatment with CIDD-99, which was not further increased in TRPC1sielnced cells. As cell viability worsens over time, so does the ability of the oral cancer cells to activate TRPC1 channel, which is critical for restoring calcium ER stores and mitochondrial function, suggesting a relationship between the two is critical for maintain oral cancer cell survival. TRPC1 channels have been shown to initiate calcium entry and have been shown to play a critical role in cancer progression (Villalobos et al., 2019; Zhang et al., 2020). Importantly, cell migration was also inhibited by the addition of CIDD-99, suggesting that loss of calcium signaling could also be important for inhibiting metastasis of oral cancer cells. Although the exact mechanism as how CIDD-99 decreases TRPC1-mediated calcium entry is not established, STIM1 expression was downregulated by the addition of CIDD-99 which could be the reason why TRPC1-mediated calcium entry was decreased. STIM1 expression is shown to be modulated post-transcriptionally by miR-223 and miR-150, however if these are altered by CIDD-99 is not known. Alternatively, decrease in ER calcium levels could also decrease the expression of TRPC1 and more research is needed to fully establish these links. Nonetheless, STIM1 is an ER resident protein and functions as an ER- Ca^2+^ sensor (Pani et al., 2009). Depletion of ER Ca^2+^ stores leads to the translocation of STIM1 to the ER-plasma membrane junctions, where it interacts with cell surface TRPC1 and Orai channels to induce Ca^2+^ influx (Pani et al., 2009; Pani and Singh, 2009). Importantly, Orai1 has also been shown to modulate TRPC1 activity (Pani and Singh, 2009); however, no change in Orai1 expression was observed in this study. In addition, the current properties observed in oral cancer cells mimicked those observed with the TRPC1 channel, suggesting that TRPC1 could be the major calcium influx channel in these cells.

The imidazole compound SKF-96365 and related antimycotic compounds has been shown to inhibit Orai and TRP channels that inhibits cancerous cell growth and tumorigenesis (Schaar et al., 2016). Our results showed that addition of SKF-96365 decreased TRPC1-mediated calcium entry in oral cancer cells and induced ER stress. Importantly, treatment of SKF-96365 has also been reported to enhance radio-sensitization in glioblastoma via cell cycle arrest (Ding et al., 2010). Our data are consistent with these findings as SKF-96365 had a synergistic effect with CIDD-99 and was able to enhance cell death in oral cancer cells; suggesting that other TRPC channels may also be involved. Similarly, TRPC1 has been shown to associate with other TRPC isoforms/regulatory proteins (Hofmann et al., 2002; Bollimuntha et al., 2005; Liu et al., 2005; Pani and Singh, 2009) and loss of these interactions could also play a critical role, and more research is needed to determine if other TRPC or its association with TRPC1 is altered by CIDD-99. Our working hypothesis is that oral cancer cells treated with CIDD-99 undergo apoptosis due to either 1) inhibition of mitochondrial function or 2) induction of ER stress, which are both modulated by calcium entry via TRPC1 channels (Singh and LiuAmbudkar, 2005; Selvaraj et al., 2012; Sukumaran et al., 2016). We demonstrate that both mitochondrial dysfunction and ER stress are results of CIDD-99 treatment. A possible explanation for this could be that CIDD-99 inhibits mitochondrial calcium signaling that could block electron transport by competing with CoQ for electrons, which led to the production of reactive oxygen species. This claim is further supported by evidence collected prior to this study which shows that the antioxidant N-acetyl cysteine is capable of reversing the anti-proliferative effects induced by CIDD-99 treatment (De La Chapa et al., 2019a). Interestingly, the effects of CIDD-99 were also reversed by increasing external calcium, which could also increase calcium in the mitochondria and increase its function. In conclusion, our data validates that CIDD-99 mediates both mitochondrial function and ER stress in oral cancer cells and provides valuable insight into a novel, cancer-selective mechanism via the calcium signaling nexus.

## Data Availability

The raw data supporting the conclusions of this article will be made available by the authors, without undue reservation.
